# Efficacy and safety of FDG-PET for determining target volume during intensity-modulated radiotherapy for head and neck cancer involving the oral level

**DOI:** 10.1186/s41824-024-00197-6

**Published:** 2024-03-15

**Authors:** Yasuo Kosugi, Keisuke Sasai, Naoya Murakami, Tatsuki Karino, Yoichi Muramoto, Terufumi Kawamoto, Masaki Oshima, Noriyuki Okonogi, Jun Takatsu, Kotaro Iijima, Shuhei Karube, Akira Isobe, Naoya Hara, Mitsuhisa Fujimaki, Shinichi Ohba, Fumihiko Matsumoto, Koji Murakami, Naoto Shikama

**Affiliations:** 1 Department of Radiation Oncology, Juntendo University Graduate School of Medicine, 2-1-1 Hongo, Bunkyo-ku, Tokyo, 113-8421 Japan; 2 Department of Radiology, Juntendo University Hospital, Tokyo, Japan; 3 Department of Otorhinolaryngology, Faculty of Medicine, Juntendo University, Tokyo, Japan; 4 Department of Radiology, Juntendo University, Tokyo, Japan; 5 Department of Radiation Oncology, Kansai Electric Power Hospital, Osaka, Japan

**Keywords:** Head and neck cancer, Intensity-modulated radiation therapy, Target delineation, 18F-FDG, PET-CT, Interobserver variability, Oral cavity, Oropharynx, Dental artifacts

## Abstract

**Purpose:**

To determine the efficacy and safety of target volume determination by ^18^F-fluorodeoxyglucose positron emission tomography-computed tomography (PET-CT) for intensity-modulated radiation therapy (IMRT) for locally advanced head and neck squamous cell carcinoma (HNSCC) extending into the oral cavity or oropharynx.

**Methods:**

We prospectively treated 10 consecutive consenting patients with HNSCC using IMRT, with target volumes determined by PET-CT. Gross tumor volume (GTV) and clinical target volume (CTV) at the oral level were determined by two radiation oncologists for CT, magnetic resonance imaging (MRI), and PET-CT. Differences in target volume (GTV_PET_, GTV_CT_, GTV_MRI_, CTV_PET_, CTV_CT_, and CTV_MRI_) for each modality and the interobserver variability of the target volume were evaluated using the Dice similarity coefficient and Hausdorff distance. Clinical outcomes, including acute adverse events (AEs) and local control were evaluated.

**Results:**

The mean GTV was smallest for GTV_PET_, followed by GTV_CT_ and GTV_MRI_. There was a significant difference between GTV_PET_ and GTV_MRI_, but not between the other two groups. The interobserver variability of target volume with PET-CT was significantly less than that with CT or MRI for GTV and tended to be less for CTV, but there was no significant difference in CTV between the modalities. Grade ≤ 3 acute dermatitis, mucositis, and dysphagia occurred in 55%, 88%, and 22% of patients, respectively, but no grade 4 AEs were observed. There was no local recurrence at the oral level after a median follow-up period of 37 months (range, 15–55 months).

**Conclusions:**

The results suggest that the target volume determined by PET-CT could safely reduce GTV size and interobserver variability in patients with locally advanced HNSCC extending into the oral cavity or oropharynx undergoing IMRT.

*Trial registration*

UMIN, UMIN000033007. Registered 16 jun 2018, https://center6.umin.ac.jp/cgi-open-bin/ctr_e/ctr_view.cgi?recptno=R000037631

## Introduction

The advent of intensity-modulated radiation therapy (IMRT) for head and neck cancer has allowed a reduction in the radiation dose to risk organs, while maintaining the dose to the target volume (TV) (Pow et al. 2006; Kam et al. 2007). However, the dose distribution is steeper and more complex in IMRT than in conventional three-dimensional (3D) conformal radiation therapy, with the possibility of marginal recurrence (Schoenfeld et al. 2008; Eisbruch et al. 2004; Raktoe et al. 2013). Inter-institutional differences in treatment outcomes and adverse events (AEs) have also increased in the era of IMRT (Boero et al. 2016), suggesting that variables in target delineation could result in differences in clinical outcomes. More accurate and standardized TV determination and reduced interobserver variability are therefore needed for IMRT planning.

TV determination using ^18^F-fluorodeoxyglucose positron emission tomography (FDG-PET) has been widely studied in patients with advanced head and neck squamous cell carcinoma (HNSCC). FDG-PET-based TVs are smaller than those obtained with computed tomography (CT) or magnetic resonance imaging (MRI), and have shown good agreement with local tumor extent determined by histopathology using surgically resected specimens (Lapa et al. 2021; Caldas-Magalhaes et al. 2012; Chatterjee et al. 2012; Daisne et al. 2004; Geets et al. 2006; Guido et al. 2009). Leclerc et al. reported that TV delineation based on FDG-PET could reduce the TV and radiation doses to the parotid gland and oral cavity, especially in patients with oropharyngeal and oral cancers (Leclerc et al. 2015). Other studies noted that TV determination by PET was particularly useful in cases with dental artifacts, suggesting that PET-based TV determination may be particularly useful in the oral cavity and oropharynx (Gardner et al. 2009; Anderson et al. 2014). Notably however, one study found no usefulness of TV determined by PET compared with MRI-based TV in patients with oropharyngeal cancer (Daisne et al. 2004). Furthermore, there is currently no uniform method for determining TV by PET. It has been suggested that TV delineation could be compromised by dental artifacts and may tend to be larger than it should be in tumors extending to the oral cavity or oropharynx, indicating the potential usefulness of PET-guided TV delineation for such tumors (Gardner et al. 2009). In the present study, we prospectively investigated the efficacy and safety of TV determination by FDG-PET using a multiple-threshold method in patients with HNSCC extending to the oral cavity or oropharynx (Okubo et al. 2010).

## Materials and methods

### Study design and data collection

We prospectively analyzed data for 10 consecutive patients with locally advanced HNSCC, in whom the primary site involved the oral level and who were treated with IMRT at Juntendo University Hospital. Extension to the oral level was defined as extension of the gross tumor volume (GTV) to the oral cavity or oropharynx. The study protocol was approved by the Juntendo hospital ethics committee (Approval no.: 18-0030), and the study was conducted in accordance with the principles of the Declaration of Helsinki. The eligibility criteria were as follows: patients aged ≥ 20 years; ECOG performance status ≤ 2; patients with locally advanced HNSCC lesions extending to the oral level with dental artifacts, including patients with de novo or locally recurrent advanced HNSCC without prior radiation therapy; and patients scheduled for definitive or postoperative radiotherapy with a total dose > 50 Gy. The exclusion criteria were patients with uncontrolled diabetes or co-morbidities deemed difficult to treat, at the discretion of the attending radiation oncologist. Staging was performed in accordance with the Union for International Cancer Control (8th edition), based on physical examination, laryngoscopic examination, CT, PET-CT, and/or MRI. For patients with recurrent disease, the stage of disease at the time of recurrence was registered. The primary endpoints were size of the TV and interobserver variability, and the efficacy of TV determination by PET compared with CT and MRI. The secondary endpoints included AEs of IMRT determined by PET and their clinical outcomes. We considered a positive result if the size of the TV and the interobserver variability were smaller with PET than those contoured based on other modalities, while maintaining acceptable local control without severe AEs, compared with previous studies of IMRT for HNSCC.

### Acquisition of images from planning CT, PET-CT, and MRI

A Type-S head and shoulder mask (CIVCO Medical Solutions, Iowa, USA) was used as the immobilization device. Planning CT (Aquilion LB, Canon Medical Systems, Tochigi, Japan) was performed with a 2-mm slice thickness. No iodine contrast agent was administered in any of the patients.

PET-CT image acquisitions were performed using a Canon Celesteion PCA-9000A (Canon Medical Systems). Patients were injected with 185 MBq (5 mCi) of FDG, left in a designated “quiet room” for an uptake period of 60 min, and then placed on a specialized flat table for radiotherapy planning. Patients, with thermoplastic masks, were placed in the supine position and the head and neck area was scanned, followed by a full-body PET-CT scan for staging purposes. About 30 min were required to complete the full-body PET-CT scan. The PET-CT images were reviewed by an experienced nuclear medicine radiologist (K.M.) and a radiation oncologist (Y.K.).

CT scans were acquired in the helical mode with a slice thickness of 2 mm and a pitch of 15 mm at 120 kV and tube current volume exposure control. All CT images were acquired using a matrix of 512 × 512 pixels. Voxel dimensions were 0.8 mm × 0.8 mm × 2.0 mm. PET images were acquired in the 3D mode using an axial field of view of 400 mm (two bed positions). The time for the one-bed position (196 mm) scan was 240 s. All PET images were acquired using a matrix of 208 × 208 pixels. The full width at half maximum at a distance of 10 cm from the center of the field of view reached 5.1 mm × 5.2 mm × 5.4 mm in the x, y, and z directions, respectively. The Fourier rebinning algorithm was combined with an ordered subsets expectation–maximization reconstruction (Daisne et al. 2004). Voxel dimensions were 2.0 mm × 2.0 mm × 2.0 mm.

Most patients underwent MRI with a 1.5-Tesla system (Avanto; Siemens, Munich, Germany) employing the 3D-gradient echo technique. The region from the skull base to the inferior margin of the sternal end of the clavicle was examined with a head and neck combined coil. After intravenous injection of gadolinium contrast agent (Prohance; Bracco Japan, Tokyo, Japan) at a dose of 0.2 mmol/kg body weight, T1-weighted fat-suppressed axial, coronal, and sagittal sequences were performed sequentially. The section thicknesses and intersection gaps were 2 mm and 0.9 mm for the axial plane, respectively.

### Volume delineation

Following all scans, the PET, CT, and MRI datasets were converted to the digital imaging and communication in medicine (DICOM) format and transferred to a radiotherapy planning system (Eclipse instrument; Varian Medical Systems, Palo Alto, CA, USA). To compare the CT-based or MRI-based TVs with the PET-based TVs, the GTV and clinical target volume (CTV) of primary lesions were contoured by two observers (K.S. with > 35 years of experience and Y.K. with > 10 years of experience in HNSCC imaging and radiation therapy) with no knowledge of the other modality images, to produce GTV_PET_, GTV_CT_, GTV_MRI_, CTV_PET_, CTV_CT_, and CTV_MRI_. The nodal GTVs and nodal CTVs were delineated on the planning CTs using international guidelines, with no aid from FDG-PET scan information (Gregoire et al. 2013). The multiple-threshold method was adopted for contouring GTV_PET_ in this study (Okubo et al. 2010). Briefly, a threshold value of 2.5 standard uptake value (SUV) was adopted for primary tumors of ≤ 2 cm, and threshold values of 35% and 20% of the maximum FDG activity of primary tumors were adopted for primary tumors of 2–5 cm and > 5 cm, respectively. Each observer decided whether to include or exclude borderline FDG uptake in close proximity to primary lesions that were suspected of accumulating FDG due to normal physiological uptake or inflammation. CTVs usually consisted of an arbitrary 10 mm margin around the GTV, with corrections made to exclude anatomical barriers such as bones, muscles, or the oropharyngeal cavity, with reference to physical examination and fiber laryngoscopy findings. Only primary lesion contours at the oral level were compared in this study. Co-registration of each image was performed carefully by one co-author (Y.K.) using fusion software on Eclipse.

For actual treatment, the CTV of the primary lesion (CTV_PET_) was divided into two categories: CTV1 was the GTV with a 5 mm margin to account for anatomic barriers, and CTV2 was the CTV1 extended by an additional 5 mm, based on international guidelines (Gregoire et al. 2018). The plan target volumes were created by adding a uniform margin of 5 mm around the CTVs. Organs at risk, such as the spinal cord, brainstem, parotid glands, oral cavity, and larynx, were contoured for all patients and planning organs at risk volumes (PRVs) were created for serial organs, such as the spinal cord or brainstem. In cases with no direct tumor invasion to the spinal cord or brainstem, the observation of dose constraints for these PRVs was prioritized over TV coverage.

### Treatment planning

The sequential boost method was employed in cases with nasopharyngeal carcinoma extending to the oropharynx, with the CTV of the primary tumor and metastatic lymph nodes irradiated with 70 Gy in 35 fractions and the prophylactic lymph node area irradiated with 50 Gy in 25 fractions. Cases with non-nasopharyngeal HNSCC were treated with the simultaneous integrated boost method, with 70 Gy to CTV1 of the primary tumor and CTV of metastatic lymph nodes, 60 Gy to CTV2, and 54 Gy to prophylactic lymph node areas in 35 fractions. IMRT was delivered with 6-MV photons using a TomoTherapy HD unit (Accuray Inc., Sunnyvale, CA, USA). The dose constraints are listed in Appendix 1.

### Analysis of recurrence

Tumor recurrences were determined by clinical examination and CT, MRI, or FDG-PET imaging. Local control (LC), progression-free survival (PFS), and overall survival (OS) were calculated from the date of study registration to the date of the event using the Kaplan–Meier method. Patients alive at the time of analysis were censored at their last follow-up visit. The location of local failure was compared with the PET-based dose distribution. The recurrent volume was defined in previous studies as: “in-field”, “extending outside the field” or “out-of-field” if it had received ≥ 95%, 20%–95%, or < 20% of the prescribed dose, respectively (Leclerc et al. 2015).

### Delineation agreement analysis

The Dice similarity coefficient (DSC) was employed as a standard and intuitive metric for comparison in this study. Computation of the DSC involved doubling the overlap volume of the two given volumes (V_overlap_) and subsequent division by the sum of the two volumes (V1, V2), as follows:

$$DSC = \frac{{2 \times V{\text{overlap}}}}{{{\text{V}}1 + {\text{ V}}2}}$$

The DSC for the target volume was calculated by two observers (Y.K. and K.S.) for each modality, with an ideal value of 1. A value > 0.6 (or 0.8) was generally deemed to be very good (Bland 2015); however, clinical interpretation was challenging due to the greater tolerance of DSC for the same absolute error in larger volumes compared with smaller volumes. In addition to DSC, we therefore also determined the pairwise Hausdorff distance (HD) to compare agreement in absolute terms, independent of volume. HD is defined as the maximum distance from a point in one set to the nearest point in another set; a higher HD between two sets indicates the existence of a pocket of dissimilarity between the two sets, while a zero HD indicates that the sets are identical (Cignoni and Scopigno 1998).

### Statistical analysis

The volumes determined for each modality and the interobserver variability (DSC, HD) were analyzed with Wilcoxon’s signed rank sum test. Factors associated with the DSC and HD of GTV, namely the primary site, stage, location of dental artifacts, distance between GTV and the dental artifacts, and size of GTV were analyzed. Fisher’s exact test and Spearman’s rank correlation coefficient test were used to analyze the correlations between the factors and the interobserver variability. AEs were assessed and documented according to the National Cancer Institute Common Terminology Criteria for Adverse Events version 4.0. AEs occurring within 3 months after treatment were defined as acute AEs. All statistical analyses were assessed at a significance level of 0.05 using JMP 12 (SAS Institute; Minato-ku, Tokyo, Japan).

## Results

### Patient characteristics and treatment

Ten consecutive patients with HNSCC who received IMRT between March 2019 and July 2021 were included in this prospective study. Eight patients underwent MRI. One patient refused to continue treatment due to severe acute radiation-induced mucositis and their treatment was terminated at 46 Gy in 23 fractions. This patient was included in the analysis of TVs but excluded from the analysis of clinical outcomes. The patients’ characteristics are shown in Table 1. All nine patients who completed treatment received a radiation dose of 70 Gy in 35 fractions. Regarding the treatment modalities, two patients received radiation alone, four received concurrent chemoradiation with cisplatin, and four received intra-arterial chemoradiation for sinonasal cancer (Kosugi et al. 2021).

**Table 1:** Patient characteristics

Characteristics	Number of patients
Age	
Median (range)	64 (54–84)
Sex	
Male	7
Female	3
Primary site	
Oropharyngeal cancer (p16-positive)	5 (1)
Nasopharyngeal cancer	1
Sinonasal cancer	4
Stage	
De novo	7
cStage III	1
cStage IV	6
Recurrent	3
rStage III	2
rStage IV	1
Treatment	
RT	2
CRT	4
IA-CRT	4
Pre-treatment imaging	
PET-CT	10
CT	10
MRI	8

RT = radiation therapy, CRT = chemoradiotherapy, IA-CRT = intraarterial chemoradiotherapy, PET-CT = positron emission tomography-computed tomography, CT = computed tomography, MRI = magnetic resonance imaging

### Clinical results

**Fig. 1:**
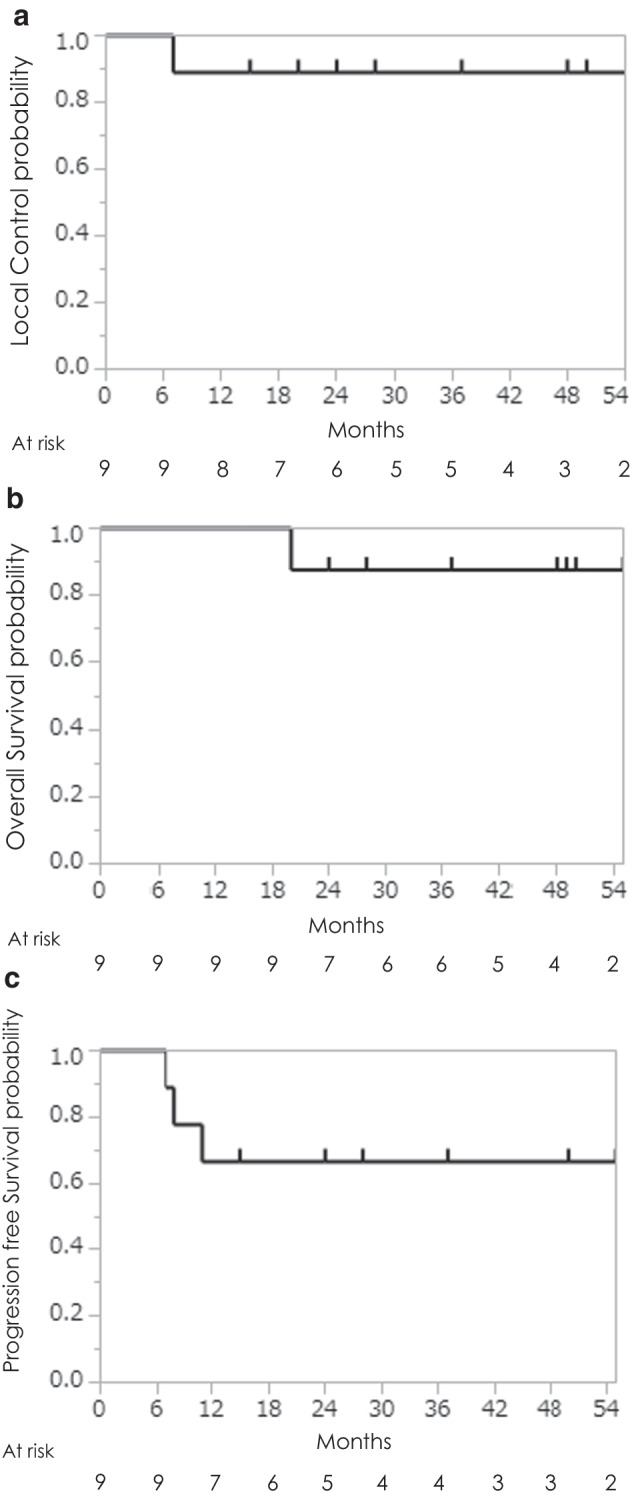
Local control rate (**a**), overall survival rate (**b**), and progression-free survival rate (**c**) following IMRT for target volume by PET-CT. White quadrangle; number of patients 10 (N = 10), gray quadrangle; number of patients 8 (N = 8)

One patient died and eight were alive after a median follow-up period of 37 months (range, 15–55 months). Two patients were lost to follow-up at 10 and 13 months, respectively, both of whom were followed up with no recurrent or metastatic lesions but were subsequently lost to follow-up because of their advanced age and difficulty in attending the hospital. The 3-year LC, OS, and PFS rates were 88%, 88%, and 67%, respectively (Fig. 1). There was one local and two regional failures. Although the local recurrence occurred ‘‘in-field’’, it was not observed at the oral level, but the sinonasal cancer progressed at the skull base. The two regional recurrences were both outside the radiation field, including one patient who underwent salvage surgery with no subsequent disease progression, and one patient who underwent palliative irradiation and subsequently died of the disease.

### AEs

The AEs associated with external radiotherapy with or without concurrent chemotherapy are summarized in Table 2. Grade ≤ 3 acute dermatitis, mucositis, and dysphagia were observed in 55%, 88%, and 22% of cases, respectively. No grade 4 AEs were observed.

**Table 2:** Acute adverse events of IMRT with target volume determined by PET-CT

Acute adverse events	Grade 2 (%)	Grade 3 (%)	Grade 4 (%)
Dermatitis	33	22	0
Mucositis	66	22	0
Dysphagia	0	22	0
Dry mouth	44	0	0
Taste disorder	44	0	0

### Volume delineation

**Fig. 2:**
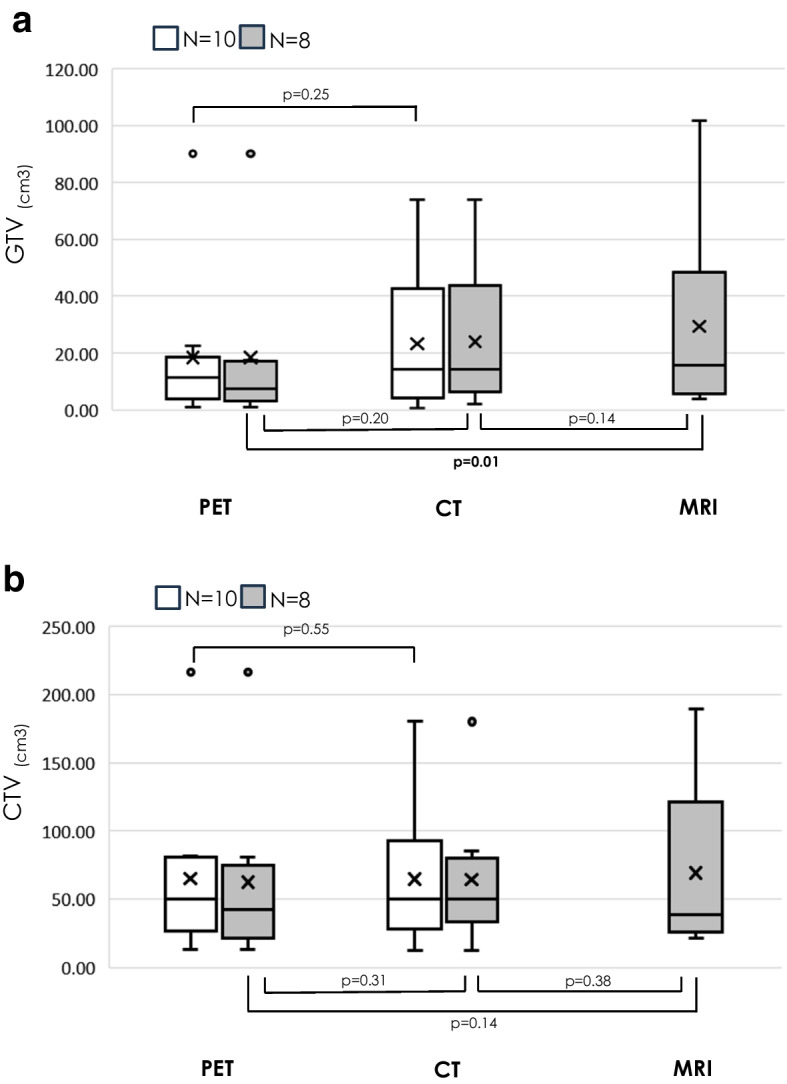
Box-and-whisker plots of GTV (a) and CTV (b) determined for each modality. The bottom and top of the boxes denote the 25th and 75th percentiles, respectively; line inside the box shows the median; crossmark shows the average; ends of the whiskers denote the maximum and minimum. Comparisons were made between each two groups with N = 10 and N = 8 comparable individuals. GTVmean (range) for each modality, GTV_PET_ was 18.3 (0.8–90.3) cm^3^ for N = 10 and 18.4 (0.8–90.3) cm^3^ for N = 8, GTV_CT_ was 23.3 (0.7–74.0) cm^3^ for N = 10 and 24.0 (1.9–74.0) cm^3^ for N = 8, GTV_MRI_ was 33.9 (4.0–101.8) cm^3^ for N = 8. CTVmean (range) for each modality, CTV_PET_ was 64.7 (13.0–216.6) cm^3^ for N = 10 and 62.5 (13.0–216.6) cm^3^ for N = 8, CTV_CT_ was 64.7 (12.2–180.0) cm^3^ for N = 10 and 64.2 (12.2–180.0) cm^3^ for N = 8, CTV_MRI_ was 86.0 (21.1–211.3) cm^3^ for N = 8

The GTV was compared in the 10 cases who underwent CT and PET-CT and in the eight who also underwent MRI. The GTV was smallest in GTV_PET_, followed by GTV_CT_ and GTV_MRI_, with a significant difference between GTV_PET_ and GTV_MRI_, but no significant difference between the other two groups. There was no significant difference between CTV_PET_, CTV_CT_, and CTV_MRI_ (Fig. 2).

### Delineation agreement

**Fig. 3:**
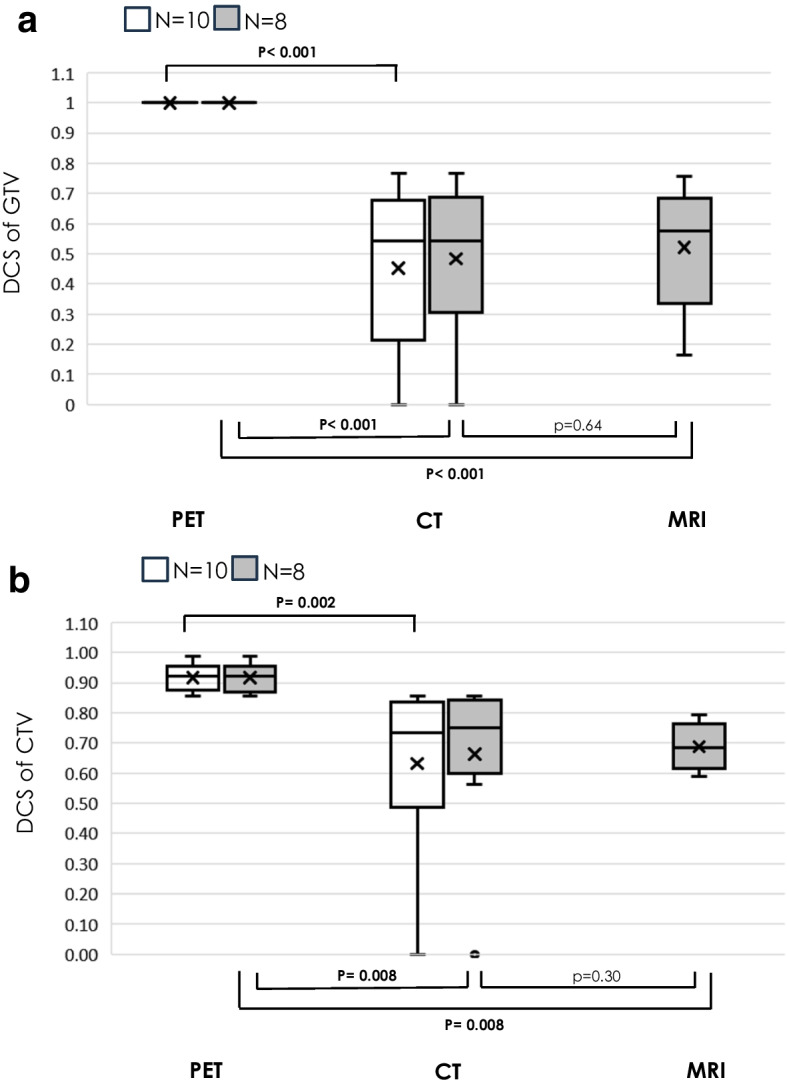
Box-and-whisker plots of DSC of GTV (**a**) and CTV (**b**) determined for each modality. Comparisons were made between each two groups with N = 10 and N = 8 comparable individuals. DSCmean (range) of GTV for each modality, DSC of GTV_PET_ was 1.0 (1.0–1.0) for N = 10 and N = 8, DSC of GTV_CT_ was 0.45 (0–0.77) for N = 10 and 0.49 (0–0.77) for N = 8, DSC of GTV_MRI_ was 0.52 (0.16–0.76) for N = 8. DSCmean (range) of CTV for each modality, DSV of CTV_PET_ was 0.92 (0.86–0.99) for N = 10 and 0.92 (0.86–0.99) for N = 8, DSC of CTV_CT_ was 0.63 (0–0.86) for N = 10 and 0.66 (0–0.86) for N = 8, DSC of CTV_MRI_ was 0.69 (0.58–0.85) for N = 8. DSC = Dice similarity coefficient

**Fig. 4:**
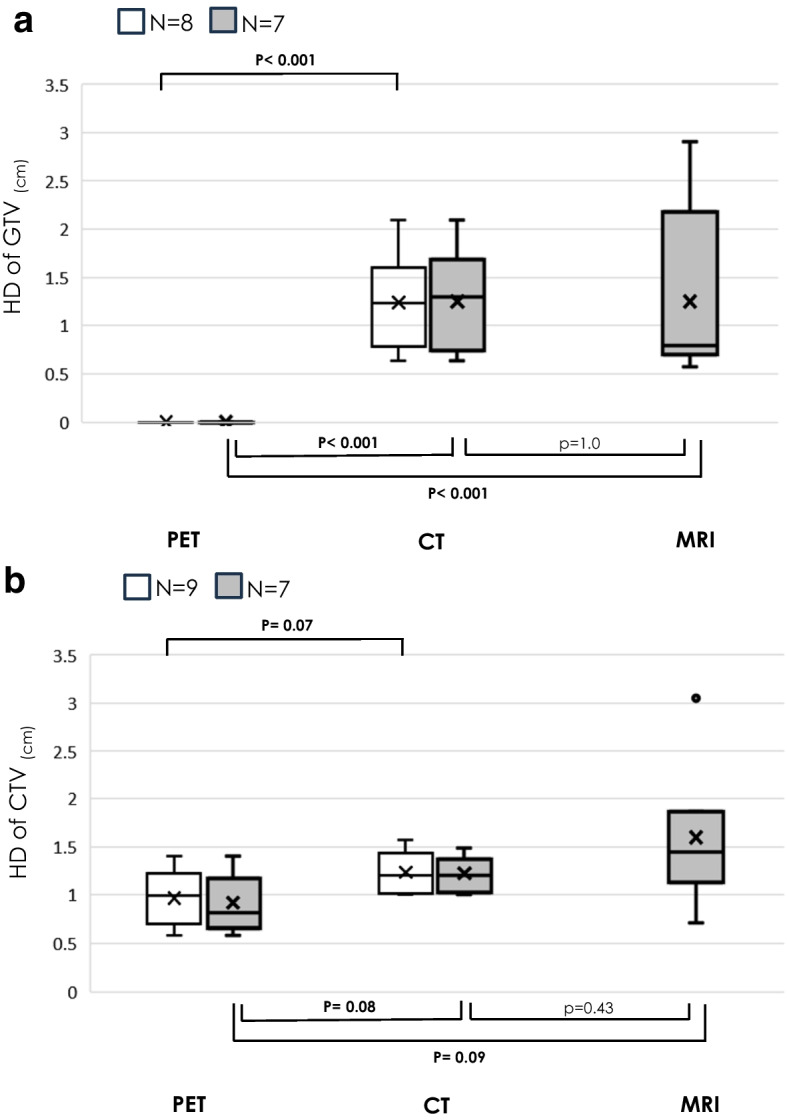
Box-and-whisker plots of HD of GTV (**a**) and CTV (**b**) determined for each modality. Comparisons were made between each two groups with N = 9, N = 8, and N = 7 comparable individuals. HDmean (range) of GTV for each modality, HD of GTV_PET_ was 0 (0–0) for N = 8 and N = 7, HD of GTV_CT_ was 1.24 (0.64–2.10) cm for N = 8 and 1.25 (0.64–2.10) cm for N = 7, HD of GTV_MRI_ was 1.25 (0.57–2.90) cm for N = 7. HDmean (range) of CTV for each modality, HD of CTV_PET_ was 0.97 (0.58–1.40) cm for N = 9 and 0.91 (0.58–1.40) cm for N = 7, HD of CTV_CT_ was 1.24 (1.00–1.57) cm for N = 9 and 1.22 (1.00–1.49) cm for N = 7, DSC of CTV_MRI_ was 1.60 (0.71–3.05) cm for N = 7. HD = Hausdorff distance

The DSC for GTV_PET_ was 1, which was significantly higher than that for GTV_CT_ or GTV_MRI_. Similarly, the DSC for CTV_PET_ was significantly higher than that for CTV_CT_ or CTV_MRI_ (Fig. 3). HD was significantly smaller for GTV_PET_ than for the other modalities, and also tended to be smaller for CTV_PET_ (Fig. 4). DSC and GTV_CT_ showed significant correlations (ρ = 0.63, P = 0.04), and HD and GTV_MRI_ also showed significant correlations (ρ = 0.96, *P* = 0.0001). The associations of DSC and HD with other factors, such as primary site, de novo or recurrent disease, and distance between GTV and dental artifacts, were not clear (Tables 3 and 4).

**Table 3:** Correlations between factors and Dice similarity coefficient in gross tumor volume

	PET			CT			MRI		
	No	DSC (median ± SD)/ correlation coefficient (ρ)	*p*-value	No	DSC (median ± SD)/ correlation coefficient (ρ)	*p*-value	No	DSC (median ± SD)/ correlation coefficient (ρ)	*p*-value
*Primary site*									
Oropharyngeal cancer	5	1	1	5	0.42 ± 0.30	0.67	3	0.63 ± 0.11	0.29
Other	5	1		5	0.49 ± 0.28		5	0.45 ± 0.23	
*Stage*									
De novo	7	1	1	7	0.44 ± 0.30	0.91	5	0.45 ± 0.23	0.3
Recurrent	3	1		3	0.47 ± 0.26		3	0.63 ± 0.11	
*Dental artifacts and GTV on the same axial plane*									
Yes	5	1	1	5	0.40 ± 0.29	0.53	4	0.60 ± 0.11	0.56
No	5	1		5	0.51 ± 0.29		4	0.44 ± 0.26	
Size of GTV					ρ = 0.63	0.04		ρ = 0.54	0.16
Distance between dental artifacts and GTV					ρ = -0.23	0.51		ρ = −0.56	0.14

PET = positron emission tomography, CT = computed tomography, MRI = magnetic resonance imaging, DSC = Dice similarity coefficient, GTV = gross tumor volume, SD = standard deviation

**Table 4:** Correlations between factors and Hausdorff distance in gross tumor volume

	PET			CT			MRI		
	No	HD (median ± SD)/ correlation coefficient (ρ)	*p*-value	No	HD (median ± SD)/ correlation coefficient (ρ)	*p*-value	No	HD (median ± SD)/ correlation coefficient (ρ)	*p*-value
*Primary site*									
Oropharyngeal cancer	4	0	1	4	1.24 ± 0.64	0.77	3	1.89 ± 1.20	0.36
Other	4	0		4	1.24 ± 0.40		5	0.73 ± 0.11	
*Stage*									
De novo	5	0	1	5	1.22 ± 0.55	0.65	5	0.73 ± 0.12	0.36
Recurrent	3	0		3	1.26 ± 0.48		3	1.88 ± 1.20	
*Dental artifacts and GTV on the same axial plane*									
Yes	4	0	1	4	1.17 ± 0.43	1	4	1.61 ± 1.10	0.3
No	4	0		4	1.30 ± 0.60		4	0.71 ± 0.13	
Size of GTV					ρ = 0.52	0.18		ρ = 0.96	0.0001
Distance between dental artifacts and GTV					ρ = −0.26	0.53		ρ = −0.31	0.44

PET = positron emission tomography, CT = computed tomography, MRI = magnetic resonance imaging, HD = Hausdorff distance, GTV = gross tumor volume, SD = standard deviation

## Discussion

Accurate TV determination and reduced interobserver variability are important factors in IMRT radiotherapy planning. For head and neck IMRT, primary site contouring guidelines have been published to equalize TV among observers (Gregoire et al. 2018); however, the availability and implementation of guidelines alone are not sufficient to ensure uniform delineation (Veen et al. 2019). The guidelines also state that FDG-PET may be useful for delineating TV in patients with locally advanced HNSCC; unfortunately however, TV determination using FDG-PET has not become common practice. We suggest two main reasons for this: first, there is currently no uniform method for determining TV using FDG-PET (Okubo et al. 2010), and second, there are few reports on the long-term safety of TV determined by FDG-PET, especially for IMRT (Leclerc et al. 2015; Matsuura et al. 2017; Wang et al. 2006; Vernon et al. 2008). Previous guidelines also stated that “PET volumes should preferably be delineated using user-independent segmentation algorithms”, but the SUV threshold for automatic TV delineation has not been standardized and various values have been proposed. Furthermore, the use of a single threshold for lesions of various sizes may increase the risk of overestimating or underestimating lesions. Hosono et al. proposed a multiple-threshold method based on lesion size to resolve the risk of a single threshold, and also reported the long-term clinical results of radiotherapy for TV determined by this method (Okubo et al. 2010; Matsuura et al. 2017). In the current study, we investigated the usefulness and safety of IMRT for TV by PET using a multiple-threshold method for lesions at the oral level, where the usefulness of TV determination by PET is controversial (Daisne et al. 2004; Leclerc et al. 2015; Okubo et al. 2010).

We performed IMRT for TV by PET in 10 patients and treatment was completed in nine patients. Regarding safety, the 3-year LC, OS, and PFS of patients who completed treatment were 88%, 88%, and 67%, respectively, which were comparable to those in previous studies of oropharyngeal cancer and maxillary sinus cancer (Eisbruch et al. 2010; Gillison et al. 2019; Homma et al. 2023). Local recurrence occurred in one patient “in-field”, but this was sinonasal carcinoma that progressed at the skull base following skull base extension before treatment, with no recurrent lesions at the oral level, and the TV determination was facilitated by PET. In addition to de novo lesions, patients with recurrent lesions were also included in this study and were treated with IMRT for TV determined by PET, with no local recurrence. This represents an important result, because there have been no previous reports on the safety of TV by PET for recurrent lesions. All patients in the present study, except for one case of nasopharyngeal carcinoma, were irradiated with high-doses of CTV1, a 5 mm extension of GTV, according to international guidelines (Gregoire et al. 2018). For recurrent cases however, the guidelines recommend extensions > 5 mm from the GTV for CTV, which is in the high-dose area due to anatomical structural disruption. TV determination by PET using a multistep method may safely reduce the high-dose irradiation area, even in recurrent cases. In the current study, the high-dose (70 Gy) irradiated area at the oral cavity level was reduced by an average of 11.7 (range: 0.3–31.2) cm^3^ in PET compared with MRI, indicating that the high-dose irradiated area was reduced by 5.9% (range; 0.1%–13.2%) of the oral cavity volume (data not shown). AEs ≤ grade 3 were less common than in previous studies of chemoradiotherapy for head and neck cancer (Gillison et al. 2019; Homma et al. 2023). Regarding TVs, GTV_PET_ was significantly smaller than GTV_MRI_, in contrast with previous findings (Daisne et al. 2004). This apparent discrepancy may be due to differences in the GTV_PET_ imaging algorithm and fusion accuracy between MRI and PET-CT. GTV_PET_ was smaller than GTV_CT_, but the difference was not significant. This may be because all patients enrolled in this study had dental artifacts, and CT images were more difficult to visualize in this group. In fact, compared with the other modalities, the interobserver variability for GTV_CT_ was larger, the DSC was smaller, and HD was larger. Given that MRI has been reported to be more useful than CT for TV determination in patients with metallic dental implants, we believe that our results provide positive information regarding TV determination by PET (Gardner et al. 2009). The results for CTV were not significantly different among the different modalities, although CTV_PET_ had the smallest volume. Previous studies of HNSCC reported that local recurrence usually occurred in the high-dose area, highlighting the need for accurate delineation of the GTV (Leclerc et al. 2015). DSC and HD, as measures of interobserver variability, showed less difference in PET than in the other modalities for both GTV and CTV. Interestingly, the DSC of GTV_PET_ was 1, indicating precise matching between the two observers. At the oral level, physiologic or inflammatory accumulation of FDG in close proximity to the GTV, specifically physiologic accumulation in the tonsils or inflammatory accumulation due to dental caries, could result in interobserver variability in GTV_PET_ (Haerle et al. 2013); however, the current results showed perfect agreement in GTV_PET_ by combining the multiple-threshold method, physical examination, and endoscopic examination. These results suggest that TV determination by PET could safely reduce the high-dose irradiation area and also reduce interobserver variability. Regarding the factors associated with DSC and HD in GTV_CT_ and GTV_MRI_, GTV showed a significant correlation, in accordance with previous studies (Veen et al. 2019). In contrast, there was no correlation with clinical stage, site of primary lesion, location of dental artifacts, or distance between the GTV and dental artifacts. This might be because the usefulness of TV determination by PET at the oral level was compounded not only by visibility due to dental artifacts, but also by the fusion accuracy between modalities due to mandibular mobility or neck curvature and anatomic complexity, which may not have been a significant factor in this limited number of cases (Gardner et al. 2009; Anderson et al. 2014). Identifying the factors responsible for the high interobserver variability of TV in CT and MRI, which may in turn make PET-CT more effective in determining TV, could help to resolve the problems of accessibility for PET-CT and limited medical resources. A larger prospective study with a unified TV delineation method by PET is needed to clarify this.

The study had several limitations. First, the number of patients was very small, and the primary sites were limited to the oropharynx, nasopharynx, and paranasal sinus among the HNSCC. Therefore, caution should be exercised when interpreting the results of this study. Second, although the single energy metal artifact reduction algorithm has demonstrated usefulness for CT metallic artifact reduction and PET-MRI with regard to cases with dental artifacts, we were unable to use it in this study (Funama et al. 2015; Huellner 2021); however, the usefulness of TV determination by PET at the oral level may be influenced by factors other than dental artifacts. Third, FDG-PET is not recommended for superficial lesions. The lack of spatial resolution of the PET camera with the partial volume effect does not allow a sufficiently accurate delineation of TV. It is therefore essential to set the TV not only by PET-CT, but also by physical examination and fiber findings. Fourth, this study did not use an iodine contrast agent in CT scans, although the international contouring guidelines recommend the use of a contrast agent (Gregoire et al. 2018); however, the greater usefulness of MRI compared with CT for TV determination has been reported in cases with dental artifacts, and we do not believe that this will affect the current results in terms of the usefulness of PET (Gardner et al. 2009; Anderson et al. 2014). Fifth, no post-treatment quality of life studies were conducted, so the impact of IMRT of the TV determined by FDG-PET using a multiple-threshold method on it is unknown. Further study is needed regarding FDG-PET-based TV determination and long-term clinical outcomes, including patients’ quality of life such as salivary gland function or swallowing function.

## Conclusions

Carrying out IMRT of the TV determined by FDG-PET using a multiple-threshold method could safely reduce the GTV and interobserver variability in patients with HNSCC lesions extending to the oral level.

## Data Availability

The data that support the findings of this study are available on request from the corresponding author.

## Appendix

See Table 5.

**Table 5:** Radiation dose constraints in this study

	Target	Permissible range
*PTV*		
Dmean	= 100% (prescribed dose)	
Dmax	< 110%	< 113%
D98	> 93%	> 90%
*CTV*		
D99	> 100%	> 98%
*Spinal cord**		
Dmax	< 50 Gy	< 54 Gy
D1cm^3^	< 46 Gy	< 50 Gy
*Brainstem*		
Dmax	< 54 Gy	< 64 Gy
D1cm^3^	< 60 Gy	
*Optic nerve*		
Dmax	< 50 Gy	< 54 Gy
*Optic chiasm*		
Dmax	< 50 Gy	< 54 Gy
*Eyeball*		
Dmax	< 40 Gy	< 45 Gy
*Lens*		
Dmax	< 6 Gy	< 10 Gy
*Parotid gland*		
Dmean	< 26 Gy	< 30 Gy
*Oral cavity*		
Dmean	< 30 Gy	< 40 Gy
*Mandible*		
D2cm^3^	< 66 Gy	< 72 Gy
*Larynx*		
Dmean	< 45 Gy	< 50 Gy
*Pharyngeal constrictor*		
Dmean	< 54 Gy	< 60 Gy

*Planning risk volume

*Dmean* mean dose; *Dmax* maximum dose; *D98* dose received by 98% of the volume; *D99* dose received by 99% of the volume; *D2cm*^*3*^ minimum dose delivered to the highest irradiated 2 cm^3^ volume; *D1cm*^*3*^ minimum dose delivered to the highest irradiated 1 cm^3^ volume; *PTV* planning target volume; *CTV* clinical target volume

## Abbreviations

IMRT: Intensity-modulated radiation therapy
TV: Target volume
3D: Three-dimensional
AEs: Adverse events
FDG-PET: Fluorodeoxyglucose positron emission tomography
HNSCC: Head and neck squamous cell carcinoma
CT: Computed tomography
MRI: Magnetic resonance imaging
GTV: Gross tumor volume
ECOG: Eastern cooperative oncology group (with reference to patient performance status)
DICOM: Digital imaging and communication in medicine
CTV: Clinical target volume
SUV: Standard uptake value
PRVs: Planning organs at risk volumes
LC: Local control
PFS: Progression-free survival
OS: Overall survival
DSC: Dice similarity coefficient
HD: Hausdorff distance
